# Association Between CKAP4 Expression and Poor Prognosis in Patients with Bladder Cancer Treated with Radical Cystectomy

**DOI:** 10.3390/cancers17081278

**Published:** 2025-04-10

**Authors:** Hiroki Katsumata, Dai Koguchi, Shuhei Hirano, Anna Suzuki, Kengo Yanagita, Yuriko Shimizu, Wakana Hirono, Soichiro Shimura, Masaomi Ikeda, Hideyasu Tsumura, Daisuke Ishii, Yuichi Sato, Kazumasa Matsumoto

**Affiliations:** 1 Department of Urology, Kitasato University School of Medicine, 1-15-1 Kitasato Minami-ku, Sagamihara 252-0374, Japan; giri_giri_zin_zin@yahoo.co.jp (H.K.);; 2 Department of Pathology, Nagaoka Chuo General Hospital, 2041 Kawasaki, Nagaoka 940-0861, Japan; 3 Biofluid Biomarker Center, Niigata University, 8050 ikarashi 2-no-cho Nishi-ku, Niigata 950-2181, Japan; 4 Kitasato University School of Medicine, 1-15-1 Kitasato Minami-ku, Sagamihara 252-0374, Japan; 5 KITASATO-OTSUKA Biomedical Assay Laboratories Co., Ltd., 1-15-1 Kitasato, Minami-ku, Sagamihara 252-0329, Japan

**Keywords:** bladder cancer, radical cystectomy, CKAP4, cancer-associated fibroblasts, immunohistochemistry, prognosis

## Abstract

**Background/Objectives**: While cytoskeleton-associated protein 4 (CKAP4) has been associated with prognosis in various malignancies, its prognostic value for bladder cancer (BCa) remains unclear. The aim of this study was to evaluate CKAP4 expression in tumor cells and cancer-associated fibroblasts (CAFs) following radical cystectomy (RC) in patients with BCa. **Methods**: In this study, CKAP4 in tumor cells was defined as CKAP4-1, while CKAP4 expressed in CAFs was defined as CKAP4-2. CKAP4-2 expression was evaluated to explore its potential association with tumor aggressiveness and patient outcomes. CKAP4 expression in 86 RC specimens was assessed using immunohistochemistry. CKAP4-1 positivity was considered when ≥5% cytoplasmic staining of cancer cells, with at least moderate staining intensity, was observed. CKAP4-2 positivity was evaluated using a point scale (0–3), with scores based on the number of CKAP4 positive CAFs in the tumor stroma. Scores of 2 (moderate number of CAFs) and 3 (significant number of CAFs) were considered to indicate positivity. **Results**: CKAP4-1 and CKAP4-2 were expressed in 53 (61.6%) and 34 (39.5%) patients, respectively. Kaplan–Meier analysis showed that patients with CKAP4-1 had significantly shorter cancer-specific survival and recurrence-free survival (RFS; *p* = 0.046 and *p* = 0.0173, respectively). Multivariate analysis showed that CKAP4-1 positivity was an independent predictor of RFS (*p* = 0.041, hazard ratio: 2.09, 95% confidence interval: 1.03–4.25). **Conclusions**: This study showed that CKAP4 expression in tumor cells may serve as a useful prognostic biomarker for patients with BCa who undergo RC.

## Simple Summary

Cytoskeleton-associated protein 4 (CKAP4), which has been linked to worse outcomes in several types of cancer, has emerged as a novel biomarker to predict patient outcomes for bladder cancer following radical cystectomy. This study investigated CKAP4 levels in bladder cancer specimens after radical cystectomy, and the association between CKAP4 levels, clinicopathological characteristics, and patient outcomes was analyzed. The analysis revealed that CKAP4 was connected to a higher risk of cancer recurrence, which means that CKAP4 could be a useful clinical tool to predict cancer recurrence after surgery.

## 1. Introduction

Bladder cancer (BCa) is the tenth most prevalent cancer globally [1]. Radical cystectomy (RC) with bilateral pelvic lymph node (LN) dissection remains the gold standard treatment for patients with muscle-invasive BCa (MIBC) and non-MIBC (NMIBC) refractory to bacillus Calmette-Guérin. Although the surgical technique has advanced and the role of pelvic lymphadenectomy is better understood, the 5-year overall survival rate after RC without neoadjuvant chemotherapy (NAC) or adjuvant chemotherapy (AC) is approximately 50% [2]. Several clinicopathological factors, such as tumor grade, lymphovascular invasion (LVI), and LN status have been studied in an effort to improve unfavorable prognoses after RC [3]. These risk factors facilitate estimation of the recurrence risk and survival outcomes; however, they do not predict individual patient prognosis. Some studies reported the efficacy of new prognostic biomarkers for BCa after RC, but none of them are available for clinical use [4,5,6]; therefore, novel prognostic biomarkers for BCa are necessary.

Cytoskeleton-associated protein 4 (CKAP4), also termed CLIMP-63 or p63, is a type II transmembrane protein with reversible palmitoylation [7,8]. It is mainly found in the rough endoplasmic reticulum, where it stabilizes its structure [9]. CKAP4 has been associated with various cancers and has attracted considerable attention in recent years. In hepatocellular carcinoma, esophageal cancer, and renal cell carcinoma, CKAP4 overexpression was significantly associated with unfavorable outcomes [10,11,12]. CKAP4 in pancreatic cancer has emerged as a potential therapeutic target for the inhibition of DKK1-CKAP4 binding and Akt activation [13]. Although the literature on CKAP4 remains scarce, a basic study has intensively investigated the mechanism of CKAP4 in the progression of BCa, indicating its potential as a novel biomarker [14]. Verification of the prognostic value of CKAP4 in clinical practice is essential for better BCa management because patients who require RC with curative intent often have poor outcomes.

The aims of this study were to evaluate the expression levels of CKAP4 in tumor cells and cancer-associated fibroblasts (CAFs) using immunohistochemical analyses of archived RC specimens and to assess the impact of CKAP4 on the prognosis of patients who underwent RC for BCa. In this study, to investigate their associations with clinicopathological features and patient outcomes, CKAP4 expressed in tumor cells was defined as CKAP4-1, while CKAP4 expressed in CAFs was defined as CKAP4-2.

## 2. Materials and Methods

### 2.1. Patients

We retrospectively analyzed the clinical data and archived specimens of patients with BCa who underwent RC with pelvic and iliac lymphadenectomies between 1990 and 2015 at the Kitasato University Hospital (Kanagawa, Japan). We also examined normal urothelial tissue specimens from adjacent tumor tissues using NMIBCs as a negative control. RC was performed in patients with pathologically proven MIBC and in those with NMIBC who failed to respond to intravesical therapy [15]. Patient characteristics were obtained from medical records, including age at RC, sex, pathological status (pT and pN stages), tumor grade, LVI status, carcinoma in situ (CIS), history of AC and salvage chemotherapy (SC), recurrence, and cancer-specific death. Pathological staging was performed according to the 2002 TNM classification. Pathological grading was performed according to the 1973 World Health Organization classification. LVI was defined as the presence of cancer cells within the endothelial space; however, cancer cells merely invading the vascular lumen were considered negative [16]. AC was performed in patients with pT ≥ 3 disease or a positive LN status. All the patients with AC or SC received platinum-based chemotherapy. Because this was a retrospective study, all patients found to be eligible during the study period were included, and no formal sample size calculation was performed. This study was conducted in accordance with the guidelines of the Declaration of Helsinki and was approved by the Ethics Committee of Kitasato University School of Medicine on 24 May 2017 (B17-010). Participants were informed of the study and provided with the opportunity to opt out at any time. As this was a retrospective study, individual written informed consent was not required.

### 2.2. Immunohistochemistry and Scoring

Three-micrometer thick sections from 10% formalin-fixed and paraffin-embedded BCa tissue blocks were deparaffinized in xylene and rehydrated in a descending ethanol series. After treatment with 3% hydrogen peroxide for 10 min, the antigen was retrieved by autoclaving in Tris-EDTA buffer (0.01 M Tris-hydroxymethyl aminomethane, 0.001 M EDTA-2Na, pH 9.0) at 121 °C for 10 min. After washing in Tris-buffered saline (TBS) for 5 min and blocking in 0.5% casein for 10 min, the sections were reacted with 1000 times diluted anti-CKAP4 polyclonal antibody (HPA000792; Sigma Life Science, St. Louis, MO, USA) for 18 h at room temperature. After rinsing with TBS three times for 5 min each, samples were treated with horseradish peroxidase-labeled polymer reagent (EnVisoin+ Dual Link System-HRP Kit; Dako, Glostrup, Denmark) for 30 min at room temperature. After rinsing with TBS three times for 5 min each, the sections were visualized with a Stable DAB solution (Invitrogen, Carlsbad, CA, USA) and counterstained with Mayer’s hematoxylin. The protocol for immunohistochemical analyses was based on a previously reported method by Yanagita et al. [17].

The expression levels of CKAP4 were evaluated in tumor cells and in CAFs located in the tumor stroma. We defined CKAP4-1 as CKAP4 in tumor cells and CKAP4-2 as CKAP4 in CAFs. CKAP4-1 was immunohistochemically evaluated by determining the intensity and percentage of positive tumor cells. Normal urothelial cells were used as an internal control. The staining intensity of the tumor cell cytoplasm was categorized into four groups: 0, no staining; 1, weak staining; 2, moderate staining; and 3, strong staining. CKAP4-1 positivity was defined as cases where ≥5% of tumor cells exhibited a staining score of 2 or 3. These scoring criteria were adapted from the study by Nagoya et al. [18]. Immunohistochemical staining for CKAP4-2 was performed based on the number of CKAP4 positive CAFs in the tumor stroma. We categorized CKAP4-2 expression into four groups: 0, no staining of CAF; 1, few CAFs; 2, moderate number of CAFs; and 3, significant number of CAFs. CKAP4-2 positivity was defined as patients with scores of 2 or 3. Because no study has evaluated CKAP4 expression in CAFs, this scoring method was adapted from a previous report by Akanda et al. [19]. Two investigators (H.K. and Y.S.) blinded to the clinical and pathological data, reviewed all the immunostained sections. Discordant cases were reviewed and discussed until consensus was reached.

### 2.3. Statistical Analyses

In immunohistochemical analysis, the age (<65 vs. ≥65), pathological stage (pT ≤ 2 vs. ≥3), LN status (N0 vs. N1 and N2), and pathological grade (1 and 2 vs. 3) were evaluated as dichotomized variables. The association between CKAP4 expression and clinicopathological status (sex, age, pathological stage, LN status, pathological grade, LVI, CIS, history of AC and SC, recurrence, and cancer-specific death) was evaluated using Fisher’s exact test. The correlation between CKAP4-1 and CKAP4-2 expression was also examined. Cancer-specific survival (CSS) and recurrence-free survival (RFS) were estimated using the Kaplan–Meier method with log-rank tests. Univariate and multivariate analyses were performed using Cox proportional hazards analysis to estimate the association between CKAP4 expression and clinicopathological variables. Statistical significance was set at *p* < 0.05. All reported *p* values are two-sided. Stata 17 for Windows (Stata, Chicago, IL, USA) was used for all analyses.

## 3. Results

The clinical data and archived specimens of 125 patients with BCa who underwent RC with pelvic and iliac lymphadenectomies were initially included. Of the total, 39 patients were excluded for the following reasons: 10 with histological variants, including squamous cell carcinoma, adenocarcinoma, and small cell carcinoma; 15 who had been previously treated with NAC; and 14 who were lost to follow-up. The final study group (n = 86) comprised 67 men (78%) and 19 women (22%). None of the patients received preoperative NAC or radiotherapy, and no distant metastases were observed at the time of diagnosis.

### 3.1. Immunohistochemistry

**Figure 1:**
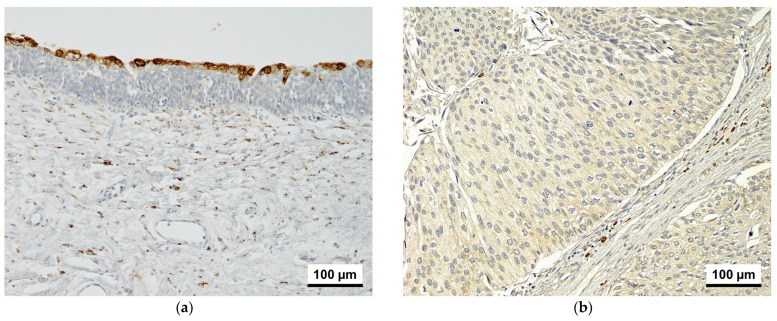

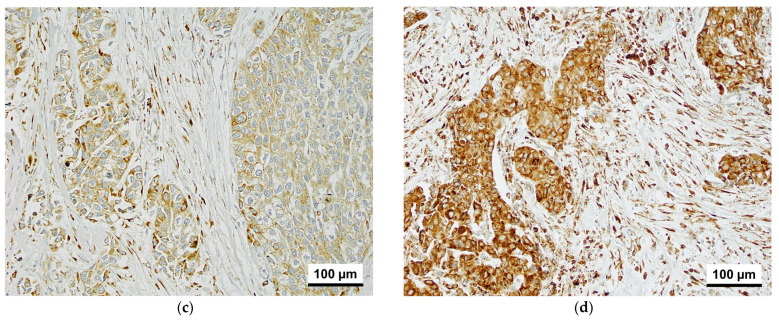
CKAP4 immunoreactivity in normal urothelium and bladder cancer tissue (200× magnification). (**a**) Normal urothelium. (**b**) Tumor cells with weak staining and a few positive CAFs. (**c**) Tumor cells with moderate staining and a moderate number of positive CAFs. (**d**) Tumor cells with strong staining and a significant number of positive CAFs. CKAP4, cytoskeleton-associated protein 4; CAFs, cancer-associated fibroblasts.

Figure 1 shows CKAP4 staining in normal urothelial and tumor tissues of the study group. In normal urothelial tissues, umbrella cells showed strong cytoplasmic staining, whereas the normal urothelium showed negative to weak staining, with scattered weakly positive normal fibroblasts (Figure 1a). In tumor tissues, CKAP4 was observed in the cytoplasm of tumor cells and CAFs at various degrees and intensities (Figure 1b–d).

### 3.2. Association of CKAP4 Expression with Clinicopathological Characteristics

Table 1 summarizes the clinicopathological characteristics of the 86 patients. CKAP4-1 and CKAP4-2 were positive in 61.6% (n = 53) and 39.5% (n = 34) of the patients, respectively. During a median follow-up of 36.0 months, the proportion of patients who died of BCa or other causes was 44.2% (n = 38) and 14.0% (n = 12), respectively. The CKAP4-1 positive group had significantly higher proportions of patients with pT ≥ 3 (67.9% vs. 39.4%) and positive LVI (72.0% vs. 48.4%) compared to the CKAP4-1 negative group. Similarly, the CKAP4-2 positive group had significantly higher proportions of patients with pT ≥ 3 (73.5% vs. 46.2%), positive LVI (84.4% vs. 49.0%), positive LN metastasis (40.6% vs. 16.0%), Grade 3 tumors (82.4% vs. 46.2%), and a history of AC (44.1% vs. 11.5%) than did the CKAP4-2 negative group.

### 3.3. Survival Outcomes and CKAP4 Expression

**Figure 2:**
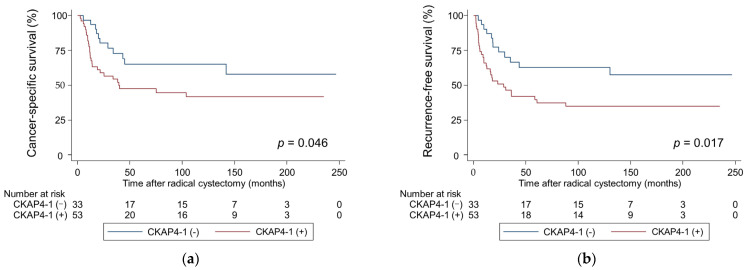
Probability of survival in patients with bladder cancer according to CKAP4-1 expression estimated using Kaplan–Meier analysis. (**a**) Cancer-specific survival; (**b**) Recurrence-free survival. CKAP4, cytoskeleton-associated protein 4.

**Figure 3:**
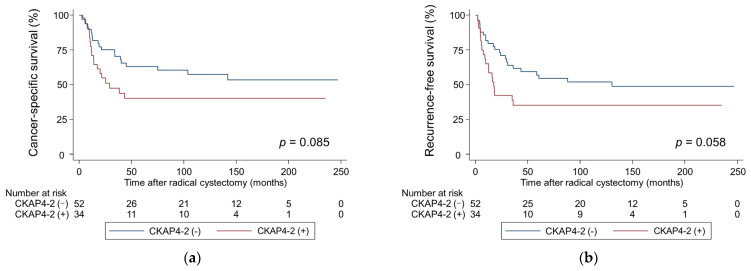
Probability of survival in patients with bladder cancer according to CKAP4-2 expression estimated using Kaplan–Meier analysis. (**a**) Cancer-specific survival; (**b**) Recurrence-free survival. CKAP4, cytoskeleton-associated protein 4.

Kaplan–Meier analysis showed that patients with positive CKAP4-1 had significantly shorter CSS and RFS (*p* = 0.046, and *p* = 0.017, respectively; Figure 2) than patients with negative CKAP4-1. Similarly, patients with positive CKAP4-2 had shorter CSS and RFS than patients with negative CKAP4-2, but the differences were not significant (*p* = 0.085 and *p* = 0.058, respectively; Figure 3). The median times to cancer death and recurrence for patients with positive CKAP4-1 were 39.3 and 28.8 months, respectively. However, for patients with negative CKAP4-1, the median times to cancer death and recurrence were not reached.

In the univariate Cox regression analysis, independent risk factors for worse CSS and RFS were pT ≥ 3, positive LN metastasis, and positive CKAP4-1. In the multivariate Cox regression analysis, positive CKAP4-1 was as an independent factor for worse RFS (hazard ratio: 2.09, 95% confidence interval: 1.03–4.25, *p* = 0.041), whereas the other clinicopathological features did not have prognostic impact regarding CSS and RFS (Table 2).

## 4. Discussion

In this retrospective analysis, we investigated the prognostic relevance of CKAP4 expression in patients with BCa who underwent RC and observed two striking findings. First, positive CKAP4 expression was associated with more aggressive pathological features, such as advanced pT stage and presence of LVI in tumor cells, and advanced pT stage, tumor grade 3, presence of LVI, and LN metastasis in CAFs. Second, the multivariate analysis adjusted for clinicopathological features revealed that CKAP4 expression in tumor cells was an independent prognostic factor for poor RFS, whereas CKAP4 expression in CAFs did not have a prognostic impact.

Although data on CKAP4 expression in BCa are scarce, pioneering work on the mechanism of CKAP4 expression in BCa cells has been reported by Sun et al. [14]. Biomarker analysis using the cell-SELEX method identified CKAP4 as having the highest affinity for the aptamer spl3, which had the best binding ability to BLCA 5637 cells. CKAP4 was involved in the progression of BCa through two potential mechanisms. First, CKAP4 facilitates cancer invasiveness by orchestrating a central-to-peripheral gradient of cell surface stiffness. For example, 5637 cells with CKAP4 exhibited a four-fold increase in motility compared to CKAP4-depleted 5637 cells, with the formation of lamellipodia in the CKAP4 positive cells presumably supporting the high migration potential. Second, exosomal CKAP4 promoted cancer metastasis. Injection of 5637 cells treated with CKAP4-containing exosome in mice exhibited approximately twice as much metastasis compared to the control 5637 cells. To assess the clinical relevance of CKAP4, the association between CKAP4 expression and the clinicopathological characteristics of patients with BCa was investigated by immunohistochemical analysis using a tissue microarray [14]. CKAP4 expression was observed in the tumor cytoplasm and nucleus and was significantly associated with MIBC and LN metastasis. As such, the mechanical properties of central-to-peripheral stiffness, lamellipodia formation, and exosomal CKAP4 significantly correlated with the invasiveness and metastasis of several cancers. In line with these findings, the worse prognostic value of CKAP4-1 demonstrated in the current multivariate analysis appears reasonable [18,20,21,22].

Another mechanism of CKAP4 underlying the biological aggressiveness of BCa is the interaction between CKAP4 and DKK1. The Wnt/β-catenin pathway plays a central role in cancer progression, and intensive research has found DKK1 as a Wnt/β-catenin pathway inhibitor [23,24]. However, recent preclinical and clinical studies have revealed that the interaction of DKK1 and CKAP4 on the cell membrane activates the Akt signaling pathway, wherein various types of cancers predominantly rely on cell proliferation [25,26]. In surgically resected esophageal squamous cell carcinoma, patients with both CKAP4 and DKK1 positivity showed significantly worse overall survival and RFS than those negative for both biomarkers [25]. Moreover, either DKK1 or CKAP4 knockdown suppressed cell growth in vitro and xenograft tumor formation via the Akt signaling pathway, and anti-CKAP4 antibody has also been demonstrated to show an antitumor effect in pancreatic cancer cells [25,26]. Although no study has reported an association between CKAP4 and DKK1, Shen et al., using western blotting, reported higher DKK1 expression levels in urothelial carcinoma tissues than in the normal urothelium [27]. The correlation between CKAP4 expression in tumor cells and poor RFS with advanced clinicopathological characteristics in the present study may represent the interaction of CKAP4 and DKK1. We believe that CKAP4 is worth highlighting as a novel prognostic and therapeutic biomarker for advanced BCa, and our findings encourage further studies to validate this hypothesis.

We observed varying levels of CKAP4 expression in CAFs within the tumor stroma, which were significantly associated with more aggressive clinicopathological features. These findings suggest that CKAP4 expression in CAFs may also be a useful biomarker for predicting the aggressive BCa phenotype. Although there are no reports on CKAP4 expression in CAFs, Gladka et al. identified CKAP4 as a novel biomarker for activated cardiac fibroblasts in a single-cell sequencing analysis, and CKAP4 was shown to be involved in myofibroblast activation in in vitro experiments [28]. Yang et al. reported the classification of CAFs into distinct subtypes that function as specific markers; in particular, myofibroblast-like CAFs (myCAFs) were found to be activated through direct interaction with cancer cells and exhibited dual tumor-restraining and tumor-promoting roles [29]. Du et al. performed an analysis using the TCGA database and reported that high expression of the myCAF marker gene was significantly associated with more advanced T-stage and worse overall survival and RFS in BCa [30]. Because myCAFs share many characteristics with myofibroblasts, it is reasonable that CKAP4 activates myCAFs similarly to myofibroblasts, promotes an aggressive phenotype of BCa and worsens survival outcomes [31,32]. However, CKAP4 expression in CAFs was not significantly associated with poor CSS or RFS. In terms of RFS, the CKAP4 positive group was significantly associated with cases that received prior AC, suggesting that CKAP4 expression did not induce poor RFS due to the treatment effect of AC. In contrast, CKAP4 positivity was not associated with SC, and the reason it did not cause significantly poorer CSS remains unclear. Although these findings are intriguing, the functional role of CKAP4 in CAFs was not analyzed in this study. Further investigations, including in vitro and in vivo studies, are needed to elucidate its biological significance.

To our knowledge, this is the first clinical study to demonstrate that CKAP4 expression in tumor tissue may be a useful prognostic biomarker for patients with BCa who undergo RC. Unlike currently available diagnostic biomarkers such as NMP22 and BTA, CKAP4 provided a novel insight into the risk of postoperative recurrence. Moreover, recent studies have reported serum or urine-based biomarkers [33,34]. In the present study, CKAP4 was evaluated in surgical specimens, and its expression can be considered to be independent of serum or urinary biomarkers. This suggests that CKAP4 could be used in combination with serum or urinary biomarkers to improve clinical decision-making.

While searching for literature on CKAP4 in PubMed and other databases, reports related to p63 were often presented in the results. Although both gene products have a molecular weight of 63,000, the CKAP4 protein is mainly localized in the cytoplasm and cell membrane [12,17,35], whereas the p63 protein is expressed in the nucleus and is known as one of the p53-related antigens [36]. Furthermore, the CKAP4 gene is located on chromosome 12q23.3 (OMIM: 618595), while the p63 gene is on chromosome 3q27-28 (OMIM: 603273). Therefore, these are different molecules. The expression of CKAP4 in human normal tissues and various tumors, as reported by The Human Protein Atlas (http://www.proteinatlas.org), is also localized in the cytoplasm and cell membrane; therefore, these proteins should not be confused with one another.

The present study had some limitations. First, it was a single-center, retrospective study with a limited sample size. Second, RC was performed by different surgeons, and the selection of postoperative chemotherapy regimens such as AC and SC depended on the preference of each doctor; this may have affected the results. Third, we did not include patients who received immune-checkpoint inhibitors or enfortumab vedotin. Future studies including these therapies may show different results. Fourth, some patient characteristics such as smoking status and occupational exposure to industrial chemicals or toxins were not included, although they may have influenced patients’ prognosis. Further studies addressing these limitations are required to validate our findings. Finally, although CKAP4 expression in CAFs was evaluated, its biological function was not investigated in this study. The functional significance should be explored using in vitro and in vivo models in future studies.

## 5. Conclusions

The expression CKAP4 in tumor cells and CAFs was significantly associated with an aggressive BCa phenotype. CKAP4 expression in tumors was an independent prognostic factor for RFS in patients with BCa who underwent RC. The findings of this study indicate that CKAP4 expression in tumors has potential as a novel biomarker for predicting tumor aggressiveness and poor prognosis in cases of BCa. Previous studies on CKAP4 in BCa are limited and involved basic research. Therefore, this clinical study using real-world data is crucial for determining the usefulness of CKAP4 as a novel clinical biomarker for BCa.

## Figures and Tables

**Table 1:** Relationship between CKAP4 and clinicopathological characteristics.

Characteristics		CKAP4-1			CKAP4-2		
	Total No. (%)	Negative (%)	Positive (%)	*p*-Value	Negative (%)	Positive (%)	*p*-Value
Overall	86	33 (38.4)	53 (61.6)		52 (60.5)	34 (39.5)	
Age, years							
Median (IQR)	65 (57–71)	65 (56–71)	64 (57–72)		65 (57–70)	64 (56–72)	
<65	42 (48.8)	15 (45.5)	27 (50.9)	0.66	24 (46.2)	18 (52.9)	0.66
≥65	44 (51.2)	18 (54.6)	26 (49.1)		28 (53.9)	16 (47.1)	
Sex							
Male	67 (77.9)	28 (84.9)	39 (73.6)	0.28	42 (80.8)	25 (75.5)	0.43
Female	19 (22.1)	5 (15.2)	14 (26.4)		10 (19.2)	9(26.7)	
pT stage							
pT ≤ 2	37 (43.0)	20 (60.6)	17 (32.1)	0.014	28 (53.9)	9 (26.5)	0.015
pT ≥ 3	49 (57.0)	13 (39.4)	36 (67.9)		24 (46.2)	25 (73.5)	
Lymph node status							
N0	61 (74.4)	26 (83.9)	35 (68.6)	0.19	42 (84.0)	19 (59.4)	0.019
N+	21 (25.6)	5 (16.1)	16 (31.4)		8 (16.0)	13 (40.6)	
Pathological grade							
G1–2	34 (39.5)	16 (48.5)	18 (34.0)	0.25	28 (53.9)	6 (17.6)	0.001
G3	52 (60.5)	17 (51.5)	35 (66.0)		24 (46.2)	28 (82.4)	
LVI							
Negative	30 (37.0)	16 (51.6)	14 (28.0)	0.037	25 (51.0)	5 (15.6)	0.002
Positive	51 (63.0)	15 (48.4)	36 (72.0)		24 (49.0)	27 (84.4)	
Carcinoma in situ							
Negative	78 (90.7)	31 (93.9)	47 (88.7)	0.70	47 (90.4)	31 (91.2)	1.000
Positive	8 (9.3)	2 (6.1)	6 (11.3)		5 (9.6)	3 (8.8)	
Adjuvant chemotherapy							
No	65 (75.6)	27 (81.8)	38 (71.7)	0.31	46 (88.5)	19 (55.9)	0.001
Yes	21 (24.4)	6 (18.2)	15 (28.3)		6 (11.5)	15 (44.1)	
Salvage chemotherapy							
No	62 (72.1)	26 (78.8)	36 (67.9)	0.32	38 (73.1)	24 (70.6)	0.81
Yes	24 (27.9)	7 (21.2)	17 (32.1)		14 (26.9)	10 (29.4)	
Recurrence							
No	43 (50.0)	21 (63.6)	22 (41.5)	0.075	29 (55.8)	14 (41.2)	0.27
Yes	43 (50.0)	12 (36.4)	31 (58.5)		23 (44.2)	20 (58.8)	
Cancer-specific death							
No	48 (55.8)	22 (66.7)	26 (49.1)	0.12	32 (61.5)	16 (47.1)	0.26
Yes	38 (44.2)	11 (33.3)	27 (50.9)		20 (38.5)	18 (52.9)	
CKAP4-2							
Negative	52 (60.5)	24 (72.7)	28 (52.8)	0.075			
Positive	34 (39.5)	9 (27.3)	25 (47.2)				

No., number; CKAP4, cytoskeleton-associated protein 4; LVI, lymphovascular invasion; IQR, interquartile range.

**Table 2:** Univariate and multivariate Cox proportional hazard analyses to predict cancer-specific survival and recurrence-free survival.

Cancer-Specific Survival							
Variables	Category	Univariate			Multivariate		
		HR	95% CI	*p*-Value	HR	95% CI	*p*-Value
CKAP4-1	Positive	2.01	0.99–4.06	0.050	1.84	0.87–3.90	0.11
	Negative	1			1		
CKAP4-2	Positive	1.74	0.91–3.30	0.089	1.24	0.56–2.73	0.58
	Negative	1					
pT stage	pT ≥ 3	2.25	1.13–4.47	0.021	1.78	0.80–3.93	0.15
	pT ≤ 2	1			1		
Pathological grade	G3	1.31	0.67–2.57	0.42	0.92	0.43–1.99	0.84
	G1–2	1			1		
LN status	N+	3.01	1.52–5.94	0.001	2.11	0.95–4.71	0.066
	N0	1			1		
CIS	Positive	0.47	0.11–1.98	0.30	0.36	0.08–1.57	0.17
	Negative	1			1		
**Recurrence-Free Survival**							
**Variables**	**Category**	**Univariate**			**Multivariate**		
		**HR**	**95% CI**	***p*-Value**	**HR**	**95% CI**	***p*-Value**
CKAP4-1	Positive	2.24	1.14–4.37	0.018	2.09	1.03–4.25	0.041
	Negative	1			1		
CKAP4-2	Positive	1.77	0.97–3.24	0.062	1.32	0.62–2.79	0.45
	Negative	1			1		
pT stage	pT ≥ 3	2.11	1.11–4.08	0.021	1.75	0.85–3.61	0.12
	pT ≤ 2	1			1		
Pathological grade	G3	1.23	0.65–2.30	0.51	0.84	0.41–1.72	0.64
	G1–2	1			1		
LN status	N+	2.71	1.42–5.16	0.002	1.85	0.88–3.87	0.10
	N0	1			1		
CIS	Positive	0.44	0.10–1.84	0.26	0.35	0.08–1.49	0.15
	Negative	1			1		

HR, hazard ratio; CI, confidence interval; CKAP4, cytoskeleton-associated protein 4; LN, lymph node; CIS, carcinoma in situ.

## Data Availability

The datasets used and/or analyzed during the study are available from the corresponding author upon reasonable request.

## Abbreviations

The following abbreviations are used in this manuscript:

CKAP4	Cytoskeleton-associated protein 4
BCa	Bladder cancer
RC	Radical cystectomy
RFS	Recurrence-free survival
LN	Lymph node
MIBC	Muscle-invasive bladder cancer
NMIBC	Non-muscle-invasive bladder cancer
NAC	Neoadjuvant chemotherapy
AC	Adjuvant chemotherapy
LVI	Lymphovascular invasion
CAF	Cancer-associated fibroblast
CIS	Carcinoma in situ
SC	Salvage chemotherapy
TBS	Tris-buffered saline
CSS	Cancer-specific survival
myCAF	Myofibroblast-like cancer-associated fibroblast
